# Nutrition and Gut Microbiota–Immune System Interplay in Chronic Diseases

**DOI:** 10.3390/nu17081330

**Published:** 2025-04-11

**Authors:** Flávio Reis, Leonardo M. R. Ferreira, Eduardo Ortega, Sofia Viana

**Affiliations:** 1 Institute of Pharmacology and Experimental Therapeutics, Faculty of Medicine, University of Coimbra, 3000-548 Coimbra, Portugal; freis@fmed.uc.pt; 2 Coimbra Institute for Clinical and Biomedical Research (iCBR), Faculty of Medicine, University of Coimbra, 3000-548 Coimbra, Portugal; 3 Center for Innovative Biomedicine and Biotechnology (CIBB), University of Coimbra, 3004-531 Coimbra, Portugal; 4 Clinical Academic Center of Coimbra (CACC), 3004-561 Coimbra, Portugal; 5 Department of Pharmacology and Immunology, Medical University of South Carolina, Charleston, SC 29425, USA; ferreirl@musc.edu; 6 Hollings Cancer Center, Medical University of South Carolina, Charleston, SC 29425, USA; 7 Immunophysiology Research Group, Instituto Universitario de Investigación Biosanitaria de Extremadura (INUBE), 06006 Badajoz, Spain; orincon@unex.es; 8 Immunophysiology Research Group, Physiology Department, Faculty of Sciences, University of Extremadura, 06006 Badajoz, Spain; 9 Polytechnic Institute of Coimbra, 3045-093 Coimbra, Portugal; 10 H&TRC—Health and Technology Research Center, Coimbra Health School, Polytechnic University of Coimbra, 3045-093 Coimbra, Portugal

Non-communicable diseases, such as obesity, type 2 diabetes mellitus (T2DM), cardiovascular diseases, allergies, and cancer are escalating global health concerns. These complex diseases can stem from overnutrition, which disrupts the gut microbiota eco-system and impairs intestinal immunity, leading to systemic chronic low-grade inflammation, also known as meta-inflammation [1]. Accordingly, there is now widespread interest in how manipulating the nutritional landscape can be used to fine-tune gut microbiota and improve overall health and well-being. This Special Issue (SI) “Nutrition and Gut Microbiota–Immune System Interplay in Chronic Diseases” delves into the intricate relationships between dietary components and nutraceuticals on intestinal microbiota, gut immunity, and distal effects on key target tissues affected in chronic diseases.

Diet profoundly influences the gut microbiota’s composition and function. Nutrients such as sugars, fats, proteins, vitamins, and secondary metabolites serve as substrates for both human metabolism and microbial fermentation, generating bacterial-derived metabolites that critically influence host immunity. For instance, short-chain fatty acids (SCFAs) derived from dietary fibers and polyphenol supplementation exhibit potent immunomodulatory and anti-inflammatory properties, while excessive saturated fats can disrupt gut microbial balance, triggering intestinal dysbiosis and driving the metabolic reprogramming of immune cells, ultimately contributing to meta-inflammation [2]. Thus, the balance and diversity of gut microbiota are crucial for maintaining intestinal barrier integrity and systemic immune homeostasis.

Dietary regimens, including intermittent fasting, caloric restriction, and elimination diets, also play a pivotal role in shaping the gut microbiota–immune system interplay, with significant implications for health and disease throughout life, including on cardiometabolic, infectious, and autoimmune disorders [3,4,5]. Likewise, gut microbiota modulators, such as prebiotics, probiotics, and synbiotics (a combination of prebiotics and probiotics), can modulate key cellular mechanisms that significantly impact the progression of immune-mediated chronic inflammatory diseases, as highlighted in this SI.

The study from Luzardo-Ocampo et al. in this SI assessed the role of prolactin receptor (PRLR) signaling in shaping gut microbiota composition during early development. Researchers compared the gut microbiota of PRLR-null (*Prlr*-KO) mice to that of wild-type (WT) counterparts (*Prlr*-WT) at the weaning stage [6]. Their findings revealed that *Prlr*-KO mice exhibited significant alterations in gut microbiota diversity and composition compared to WT mice. Notably, relative abundances, phylogenetic diversity, and bacterial concentrations were lower in the *Prlr*-KO mice, with two genera (Anaerotruncus and Lachnospiraceae) related to metabolic disease development being the most common in the *Prlr*-KO mice. In addition, a higher metabolism of terpenoids and polyketides was found in the *Prlr*-KO mice, versus the control *Prlr*-WT, and these metabolites had antimicrobial properties and were present in microbe-associated pathogenicity. These microbial shifts were associated with changes in body weight and intestinal morphology, suggesting that PRLR signaling plays a crucial role in establishing a healthy gut microbiota in early life. The study highlights the importance of maternal prolactin in modulating offspring gut health, potentially influencing susceptibility to metabolic disorders later in life.

Wu et al. investigated the impact of long-term fasting on gut microbiota composition and serum metabolome in 13 adult men [7]. The findings revealed that 10-day complete fasting significantly altered gut microbiota diversity and composition, increasing the Proteobacteria phylum approximately six-fold, while decreasing Bacteroidetes by about 50% and Firmicutes by 34%. Microbial shifts were associated with changes in serum metabolites, suggesting a link between prolonged fasting, gut microbiota alterations, and host metabolic profiles. Notably, Ruthenibacterium lactatiformans was found to be highly increased during long-term fasting, presenting a robust correlation with fat metabolic indicators. In animal studies, Ruthenibacterium lactatiformans was able to reduce high-fat diet-induced obesity, glucose intolerance, dyslipidemia, and intestinal barrier dysfunction. The clinical study of Wu et al. highlights the potential of long-term fasting as a modulator of gut microbiota and systemic metabolism, offering insights for therapeutic strategies targeting metabolic health [7]. Ruthenibacterium lactatiformans could be viewed as a probiotic candidate for the amelioration of dyslipidemia and for mediating the benefits of fasting on fat metabolism.

The review article by Mafra et al. explores the intricate connection between diet, gut microbiota, and lifestyle-related diseases [8]. The authors highlight that gut microbiota composition is significantly influenced by dietary habits and other environment factors, which in turn affect the host’s metabolism and immune system. Examining how animals adjust their gut microbiota throughout various life stages and in response to extreme environmental conditions can offer crucial insights into the role of microbiota in shaping host biology. These findings from the natural world may contribute to the development of treatments or preventive strategies for human diseases. The authors propose that knowledge derived from animal models can help design nutritional interventions to regulate gut microbiota, potentially preventing or alleviating lifestyle-related diseases in humans [8].

Focusing on the intricate relationship between diet, gut microbiota, and obesity, the review article from Patloka et al. emphasizes how dietary patterns significantly influence gut microbiota composition, which in turn affects metabolic health and obesity risk [9]. High-fat and high-sugar diets are associated with reduced microbial diversity and an increase in pro-inflammatory bacteria, contributing to metabolic disturbances. Conversely, diets rich in fiber and resistant starch, such as the Mediterranean diet, promote a diverse and balanced microbiota, enhancing metabolic functions and reducing inflammation. The review further discusses how the products of the gut microbiome metabolism, such as SCFAs and secondary bile acids, affect gut microbiota, intestinal barrier function and immune homeostasis in the framework of obesity [9]. The insights underscore the importance of considering gut microbiota in dietary approaches to prevent and manage obesity.

Using hyperinsulinemic–euglycemic clamps, Warmbrunn et al. assessed insulin resistance in 97 treatment-naive men with metabolic syndrome (MetSyn) and identified circulating biomarkers using machine learning techniques. They then validated the findings in a cohort of 282 obese individuals, both with and without MetSyn, which were analyzed through nuclear magnetic resonance imaging of adipose tissue [10]. The authors found that insulin resistance in MetSyn is associated with changes in inflammatory proteins, including IL-1, TNF receptors, and IGFBP-2, which were able to allow distinction between insulin-resistant and insulin-sensitive individuals and correlation with impaired fasting glucose, liver fat, and visceral adipose tissue. Notably, these associations were stronger in individuals with MetSyn than in obese individuals without MetSyn. The study highlights how specific proteins linked to inflammation and insulin signaling contribute to insulin resistance in MetSyn, providing potential targets for future interventions, in order to mitigate the global burden of insulin resistance and diabetes.

Ferreira et al. examined the effects of blueberry juice supplementation during 8 weeks on hepatic lipid accumulation, endoplasmic reticulum (ER) stress, and autophagy in a rat model of prediabetes induced by a high-fat, high-sucrose diet [11]. Although blueberry juice supplementation improved glucose tolerance, it led to increased serum and liver triglyceride levels, reduced thermogenesis markers in iBAT, suppressed the ER stress response, and inhibited autophagy in the liver of prediabetic rats, together with reduced SCFAs fecal content, without affecting body weight gain or adiposity. Given the crucial role of thermogenesis, hepatic autophagy, ER stress response, gut microbiota, and SCFA composition in lipid regulation, these changes suggest that blueberry juice may have detrimental effects on these mechanisms, contributing to a worsening lipid profile. Future studies should aim to determine the precise causes of these metabolic disruptions in the liver and adipose tissue and to assess the specific impact on gut microbiota. This will help clarify whether these effects could influence disease progression when blueberry juice is consumed at the prediabetic stage.

The randomized controlled pilot study of Horvath et al. explored alterations in the gut–lung axis following severe COVID-19 infection and assessed the potential modulatory effects of probiotics [12]. Their study revealed significant and lasting alterations in clinical, microbiome, metabolomic, and immune aspects of the gut–lung axis in patients recovering from severe COVID-19, with some of these changes being partially influenced by a multi-species probiotic. Specifically, when compared to COVID-19 patients after mild disease, the severe ones showed lower microbial richness, which was significantly improved by probiotic intervention; in addition, a reorganization of Ruminococcaceae and Lachnospiraceae taxa was observed in severe patients but remained unaffected by the intervention. The findings of this study emphasize notable disruptions within the gut–lung axis; however, future research must carefully consider key confounding factors, including viral load, medication use, recurrent or secondary infections, and comorbidities such as obesity and metabolic syndrome [12]. These results lay the groundwork for further investigations into acute viral infections and strategies for preventing or treating post-acute COVID-19 syndrome.

Hinchado et al. evaluated the effects of synbiotic supplementation on the quality of life and immunoneuroendocrine responses in patients with fibromyalgia (FM), some of which were co-diagnosed with chronic fatigue syndrome (CFS) [13]. This 30-day pre-/post- intervention study involved 15 FM patients, half of whom also had CFS. Results indicated that synbiotic supplementation significantly reduced perceived stress, anxiety, and depression levels, while enhancing quality of life in daily activities. Additionally, synbiotic intake led to physiological activation of the hypothalamic–pituitary–adrenal (HPA) axis, evidenced by normalized cortisol release, which in turn may counteract the elevated baseline inflammation, marked by increased interleukin-8 (IL-8) levels, in FM patients. Importantly, no adverse changes in body composition, sleep parameters, or activity levels were noted. These findings clearly suggest that synbiotic supplements can improve the disrupted immune–neuroendocrine interaction and reduce stress, anxiety, and depression, improving quality of life in women with FM, especially those without a prior CFS diagnosis [13].

In conclusion, the pre-clinical and clinical studies reported in this SI underscore the crucial role of diet on gut microbiota and immune function across different life stages and health conditions. Future research should advance personalized nutrition strategies tailored to individual microbiota compositions and genetic backgrounds. A deeper understanding of the intricate diet–microbiota–immune system interactions opens new avenues for nutritional interventions for prevention and management of chronic diseases, paving the way for innovative dietary guidelines and nutraceutical approaches.
